# Hybrid feature optimized CNN for rice crop disease prediction

**DOI:** 10.1038/s41598-025-92646-w

**Published:** 2025-03-06

**Authors:** S. Vijayan, Chiranji Lal Chowdhary

**Affiliations:** School of Computer Science Engineering and Information Systems, Vellore Institute of Technology, Vellore, 632014 India

**Keywords:** Hybrid-optimization, Feature extraction, Crop, CNN, Bio-inspired optimization, Whale optimization, Adaptive particle swarm optimization, Computational science, Computer science, Information technology

## Abstract

The agricultural industry significantly relies on autonomous systems for detecting and analyzing rice diseases to minimize financial and resource losses, reduce yield reductions, improve processing efficiency, and ensure healthy crop production. Advances in deep learning have greatly enhanced disease diagnostic techniques in agriculture. Accurate identification of rice plant diseases is crucial to preventing the severe consequences these diseases can have on crop yield. Current methods often struggle with reliably diagnosing conditions and detecting issues in leaf images. Previously, leaf segmentation posed challenges, and while analyzing complex disease stages can be effective, it is computationally intensive. Therefore, segmentation methods need to be more accurate, cost-effective, and reliable. To address these challenges, we propose a hybrid bio-inspired algorithm, named the Hybrid WOA_APSO algorithm, which merges Adaptive Particle Swarm Optimization (APSO) with the Whale Optimization Algorithm (WOA). For disease classification in rice crops, we utilize a Convolutional Neural Network (CNN). Multiple experiments are conducted to evaluate the performance of the proposed model using benchmark datasets (Plantvillage), with a focus on feature extraction, segmentation, and preprocessing. Optimizing feature selection is a critical factor in enhancing the classification algorithm’s accuracy. We compare the accuracy, sensitivity, and specificity of our model against industry-standard techniques such as Support Vector Machine (SVM), Artificial Neural Network (ANN), and conventional CNN models. The experimental results indicate that the proposed hybrid approach achieves an impressive accuracy of 97.5% (Refer Table 8), which could inspire further research in this field.

## Introduction

Rice crop is a staple meal for more than half of the world’s population, so one of the most important staple crops in the world is rice^1^. Numerous illnesses impact rice harvests, resulting in significant reductions in output. Different diseases have different symptoms in terms of size, color, form, and other attributes. Certain infections might have patterns that are similar in color but have various tones. Some are colored differently yet in the same state. When selecting pesticides, farmers usually lack clarity and are unable to make decisions^2^. Diseases that affect rice crops, like Brown Spot, Hispa, and Leaf Blast, can result in severe losses if they are not identified and treated promptly^3^. However, a number of diseases can affect rice harvests, ensuring food security and enhancing crop management techniques depend on the promptly detection and diagnosis of these diseases.

The conventional method that is manual visual inspection process used in traditional illness detection methods is labor-intensive, prone to human error, and necessitates specialized knowledge^4^. Crop diseases directly control the crops yield. In order to improve rice crop disease identification, automation of the disease identification process appears to be a promising use of recent advances in ML, DL and image processing techniques. Automated agricultural disease identification has been made possible by recent developments in artificial intelligence, notably in the fields of computer vision, ML and DL^5^.

**Fig. 1:**
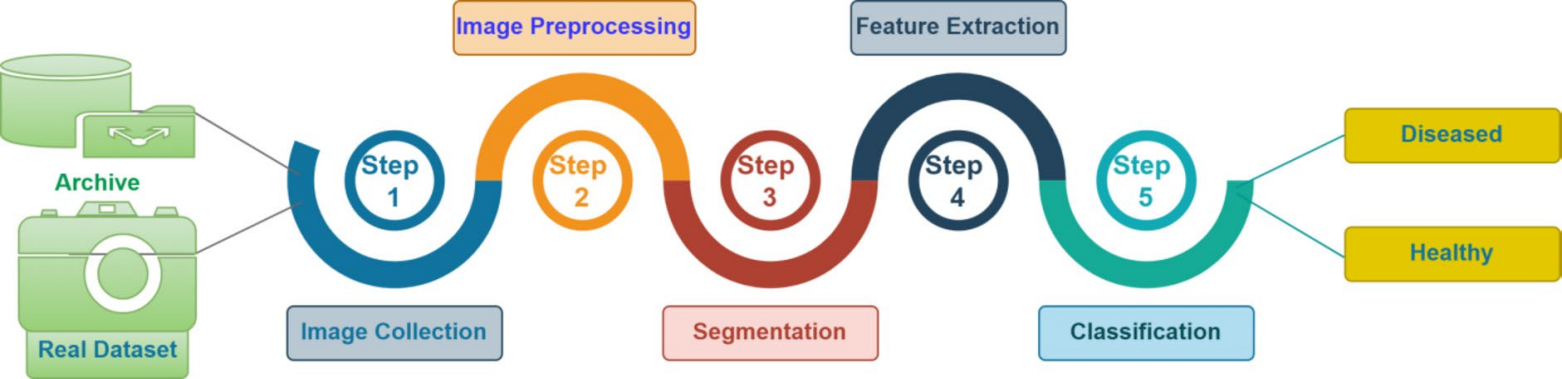
Basic image classification process.

The Fig. 1 shows that basic image classification process. The initial process of image classification process is collect the crop images, the crop image we may collect form the existing image depository or collected from the field, followed by image pre-processing. Normalization and enhancement are two common image pre-processing techniques used to raise image quality and lower distortion. The common pre-processing includes resizing, gray scaling, noise reduction, normalization, binarization, contrast enhancement.

The different kinds of filters are used in the frequency and spatial domains for image enhancing techniques^6^. The wiener filter uses a high pass filter (inverse filtering) to perform deconvolution on the rice crop diseases image dataset and a low pass filter to reduce noise^7^. In order to properly extract the features from the affected region of a crop disease image, segmentation is the most challenging process. Many segmentation methods exist, including region based segmentation^8,9^ and watershed transform edge based segmentation^10^ Mask R-CNN^11^. The technique utilized for feature extraction in crop imaging includes Gray-level o-occurrence matrix (GLCM), Gray-level run-length matrix (GLRLM), Histogram features, Gray-level dependence matrix (GLDM), and Local binary pattern (LBP) because there are notably more characteristics in this type of image^12,13^.

Authors in^14^ introduced the CNN can directly capture spatial hierarchies of features from raw data, it is used for automatic feature extraction. Boruta feature selection algorithms are used in order to identify the statistically relevant features. These collections of features that have been derived from crop imagery offer important information that aids in analyzing pat terns and making decisions. Furthermore, by choosing the most important characteristics, feature selection and optimization strategies can improve classification model performance by lowering computing complexity and increasing accuracy.

Authors in^15^ proposed a customised CNN for maize plant disease identification, the model is demonstrate with a array of preprocessing techniques, including log transformation, RGB to HSV (Hue, Saturation, and Variance) conversion of images, and Contrast Limiting Adaptive Histogram Equalization (CLAHE) on each RGB (Red, Green, and Blue) channel. The trained models are contrasted with CNN and SVM that were not pre-processed. They were using the PlantVillage maize crop dataset in order to assess the efficacy of the suggested work. A maximum accuracy of 96.76% is attained in this work.

The proposed work attempts to create a solid methodology that combines a deep learning strategy with a bio-inspired hybrid optimization algorithm. In order to optimize feature selection, we present in this study a hybrid bio-inspired algorithm called WOA_APSO that combines the WOA and APSO. Our approach attempts to create a precise and computationally efficient solution for rice crop disease detection by incorporating CNNs for disease classification.

### Motivations and contributions

#### Motivations

The whale optimization algorithm was developed as a metaheuristic algorithm with many good properties, such as few parameters, covering a larger search space, and efficient search space exploration through the use of randomly selected search agents instead of the best search agent to date. The latest development in the field of optimization is called “metaheuristic hybridization” which is accomplished by fusing two complementary metaheuristic algorithms^16–19^. Hybrid bio-inspired WOA_APSO, a novel variation of WOA and APSO, are suggested for the purpose of choosing optimum features.

#### Contribution

- First, we provide a smart segmentation technique for identifying unhealthy areas in crop leaf images and making predictions of crop disease.
- Second, we demonstrate the hybridization of the APSO and WOA metaheuristic algorithms. The hybrid WOA_APSO technique is used to choose the subset of optimal features. Here, linear discriminant analysis is embedded to do feature selection grouping. A CNN is used in our suggested model to aid with classification.
- Thirdly, a number of computer simulations are run in order to assess the viability of the suggested model. A two-way performance comparison was shown. The performance of the suggested model is first assessed in comparison to many cutting edge classification methods, with the metrics of accuracy, sensitivity, and specificity being assessed. Second, based on the computational cost of convergence to the optimal outcomes, the suggested algorithm’s performance is contrasted with that of the WOA and APSO standard algorithms.

#### Organization

The format of the paper is as follows: In Sect. 2 describe the various researchers, applied techniques for pre-processing, segmentation, features extractions and classification of crops disease identification and classification in the title of related work. The Sect. 3 narrate the process of developing a new model for rice crop disease identification and classification with the sub heading data collection, pre-processing, feature extraction, feature optimization in the title of proposed methodology. Section 4 wraps up the conclusion and makes some recommendations for more research.

## Related work

This section presents the relevant research and findings. Early diagnosis of agricultural diseases allows for preventative measures and improves the crop output rates. The field of crop disease study is expanding quickly to identify and classify rice crop disease, demonstrating the advancement of computationally intelligent systems. One of the most promising areas for creating a reliable expert system utilizing computer vision and artificial intelligence is data visualization for rice crop picture analysis. The use of machine learning algorithms for the identification of crop diseases has been the subject of numerous studies. The CNNs can automatically extract information from images, they are commonly employed for image classification tasks, also CNN may be used to accurately diagnose plant diseases from photos. Similar to this, CNNs were used by to diagnose illnesses in cassava plants with a high degree of accuracy^20–22^. By optimizing feature selection, bio-inspired optimization techniques have also been used to improve classification models.

The WOA has demonstrated notable success in resolving optimization issues by imitating the hunting behavior of humpback whales. Like most optimization algorithms, WOA might, however, run into issues with local exploitation, which occasionally prevents it from finding exact answers. An enhanced version of particle swarm optimization, known as APSO, modifies the behavior of the swarm in response to changing circumstances. The most difficult task in the interpretation of the decision-making process for extracting the irregularities in the image is the computer aided diagnosis of rice crop disease^23–25^.

### Image preprocessing techniques

The rapid advancement of machine learning and Artificial Intelligence (AI) has made it possible to use image processing and classification techniques to automatically detect plant illnesses. One type of deep learning models that has shown great efficiency in image-based tasks including segmentation, object detection, and classification is CNNs. CNNs function well in identifying patterns linked to various agricultural diseases because they can automatically extract pertinent information from images. CNNs have shown successful in detecting agricultural diseases, as evidenced by recent research like where their accuracy rates outperform those of more conventional machine learning models like SVMs and k-Nearest Neighbors (k-NN). In particular for small datasets, augmentation techniques such geometric alterations and intensity fluctuations aid in dataset expansion and enhance model generalization^26,27^.

In order to segment the diseased content form rice crop image author in^28^ suggested a hybrid segmentation technique that combines the region expanding algorithm with fuzzy C-means (FCM). The segmented diseased region’s statistical, geometrical, and textural features are retrieved, and a cuckoo search method is used to choose the best features. A SVM is used to evaluate the classification while taking into account the best attributes. Author in^29^ established the exponential k_best_ gravitational search strategy and proposed a methodology for multilayer picture thresholding for segmentation.

CNNs have been effectively used for illness identification in several recent researches; nevertheless, they frequently face challenges when working with limited, unbalanced datasets or when they must function in real time under unpredictable field settings. For example, real-time processing in agricultural fields is critical, but U-Net-based segmentation models’ high accuracy is limited by their computing. Furthermore, while hybrid bio-inspired algorithms like as WOA and APSO show promise in feature selection, their implementation is difficult without accurate parameter tweaking, particularly when applying to various crop kinds or datasets^30^.

### Feature extraction techniques

In order to convert processed crop images into useful information that can be applied to classification, feature extraction is essential. Texture-based characteristics have been extracted using conventional methods like the GLCM, which have been used extensively. The study of^31^ shows that GLCM is effective at capturing the textural patterns found in photographs of damaged leaves. According to^32^ shape descriptors and color moments are also frequently used to extract geometric and color-based information. Color features and mathematical expression has represented in Tables 1 and 2 representing the texture features (GLCM) and mathematical expression, Table 3 contains the statistical features and mathematical representation and Table 4 shows the geometrical features and concern mathematical expression. Using pre-trained deep learning models like VGG16, ResNet, and InceptionNet which automatically extract hierarchical features from unprocessed image dataset recent research has integrated deep feature extraction. These models’ capacity to recognize intricate visual patterns makes them excellent feature extractors that greatly increase classification accuracy deep feature extraction (ResNet, InceptionNet, VGG16) accuracy is increased and the requirement for manual feature engineering is decreased when pre-trained deep learning models automatically extract high-level features from pictures. These models work particularly well at capturing intricate patterns in images. The adoption of deep learning models, such CNNs, is restricted in resource-constrained situations like rural agricultural areas since they demand high performance GPUs for training and inference^33^.

Principal component analysis (PCA), which shrinks the feature space while maintaining a sizable variation in the data, is one dimensionality reduction technique that is necessary because feature extraction from such high-dimensional data still presents difficulties. Extraction techniques for high dimensions, particularly those involving deep learning models such as ResNet, necessitate substantial computer power. Because of this, managing massive, real-time datasets without the use of dimensionality reduction techniques is difficult^34^. The GLCM, is a tried-and-true technique for obtaining texture features. It is especially helpful in identifying textures in rice leaves that are peculiar to a given illness. It offers useful and comprehensible texture data. Conventional feature extraction methods, such as GLCM, are less scalable for big datasets since they still need manual adjustment and knowledge^39^.

**Table 1:** Color features-color moments and mathematical representations.

Ref	Name of the feature	Concern mathematical representations
^35–38^	Mean	$\:Mean=\:\sum\:_{m}^{i=1}\frac{1}{M}{P}_{ji}$
	Standard deviation	$\:StandardDeviation=\:\sqrt{\frac{1}{M}{\sum\:}_{M}^{i=1}{({P}_{ji}-{M}_{j})}^{2}}$
	Skewness	$\:Skewness=\:\sqrt[3]{\frac{1}{M}{\sum\:}_{M}^{i=1}{({P}_{ji}-{M}_{j})}^{3}}$

**Table 2:** Texture features–GLCM and mathematical representations.

Ref	Name of the feature	Concern mathematical representations
^35–38^	Entropy	$\:Entropy=\:-\sum\:\sum\:q(i,j)\text{log}q(i,j)$
	Contrast	$\:Contrast=\:\sum\:\left({i,j)}^{2}q\right(i,j)$
	Correlation	$\:Correlation=\:\frac{{\sum\:}_{i=0}^{M-1}{\sum\:}_{j=0}^{M-1}\left(i-{n}_{j}\right)q(i,j)}{{\sigma\:}_{i}{\sigma\:}_{j}}$
	Energy	$\:Energy=\:\sum\:\sum\:q{(i,j)}^{2}$
	Homogeneity	$\:Homogeneity=\:\sum\:_{i,j}\frac{q(j,i)}{1+|\:j-i\:|}$
	Coarseness (tissue largest size)	$\:{R}_{M\left(x,y\right)=\:{\sum\:}_{i=x-{2}^{M-{1}_{-1}}}^{x+{2}^{M-1}}{\sum\:}_{j=y-{2}^{M-{1}_{-1}}}^{y+{2}^{M-1}}\frac{F(i,j)}{{2}^{2M}}}$
	Coarseness	$\:{S}_{M,h}\left(x,y\right)=|\:{R}_{M}\left(x+{2}^{M-1},y\right)-{R}_{M}(x-{2}^{M-1},y\:|$
	Contrast	$$\:Contrast=\:\raisebox{1ex}{$\sigma\:$}\!\left/\:\!\raisebox{-1ex}{$\alpha\:4$}\right.\:,\:\:{\alpha\:}_{4}=\:\raisebox{1ex}{${N}_{4}$}\!\left/\:\!\raisebox{-1ex}{${\sigma\:}^{4}$}\right.$$
	Directionality	$\:Directionality=1-{rm}_{peaks}{\sum\:}_{p=1}^{{m}_{peaks}}{\sum\:}_{b\in\:{w}_{p}}{\left(b-{b}_{p}\right)}^{2}{H}_{directionality}\left(b\right)$
	Line – likeness	$\:flin=\sum\:\sum\:PDd\left(i,j\right)njmicos\left[\left(i-j\right)\right]2\pi\:n]\sum\:\sum\:PDd\left(i,j\right)njmi$
	Regularity	$\:{Y}_{Regularity}=1-R({C}_{CRS}+{C}_{con}+{C}_{dir}+{C}_{lin})$
	Roughness	$\:Roughness=Coarseness+contrast$

**Table 3:** Statistical features and mathematical representations.

Ref	Name of the feature	Concern mathematical representations
^35–38^	Contrast	$\:Contrast\left(F\right)={\sum\:}_{m}^{{M}_{{g}^{-1}}}{m}^{2}\left[{\sum\:}_{i=0}^{{M}_{{g}^{-1}}}{\sum\:}_{j=0}^{{M}_{{g}^{-1}}}qd,\theta\:\:(i,j)\right]$
	Entropy	$\:Entropy=\:-sum(P*\text{log}\:\left(P\right))$
	Root Mean Square	$\:RMS=\:\sqrt{\frac{1}{M}{\sum\:}_{j=1}^{M}|{y}_{j}{|}^{2}}$
	Energy	$\:Energy\left(F\right)=\:\sum\:_{j}\sum\:_{i}q{(j,i)}^{2}$
	Kurtosis	$\:Kurtosis=\:\frac{{\sum\:}_{i}{\sum\:}_{j}q\left(i,j\right)-{M}_{x}{M}_{y}}{{\sigma\:}_{x}{\sigma\:}_{y}}$
	Variance	$\:Variance\:\left({\sigma\:}^{2}\right)=\frac{1}{q}\sum\:_{i=1}^{q}{({Y}_{j}-M)}^{2}$
	Central movement	$\:5thcentralmovement=\:{\sum\:}_{j=1}^{M}{\sum\:}_{i=1}^{N}\frac{{(q\left(j,i\right)-m)}^{5}}{{\left(MN\right)\sigma\:}^{5}}$
	Central movement	$\:6thcentralmovement=\:{\sum\:}_{j=1}^{M}{\sum\:}_{i=1}^{N}\frac{{(q\left(j,i\right)-m)}^{6}}{{\left(MN\right)\sigma\:}^{6}}$
	Smoothness	$\:Smoothness\left(Q\right)=\:1-\frac{1}{1+{\sigma\:}^{2}}$
	Mean	$\:Mean=\:{\sum\:}_{i=1}^{r}{\sum\:}_{j=1}^{t}\frac{q(i,j)}{rt}$
	Standard deviation	$\:standarddeviation=\:\sqrt{{\sum\:}_{i=1}^{r}{\sum\:}_{j=1}^{t}\frac{{\left(q\right(i,j-m)}^{2}}{rt}}$

**Table 4:** Geometrical features with mathematical representations.

Ref	Name of the feature	Concern mathematical representations
^35–38^	Area	$\:Area=\:\frac{1}{2}\sum\:_{i=0}^{m-1}(pi*{q}_{i+1)}-\:({p}_{i+1}*{q}_{i)}$
	Slope	$\:Slope=\:\frac{pz-po}{qz-qo}$
	Perimeter	$\:Perimeter=n*\left(x\right)$ $\:Perimeter=\:{\sum\:}_{i=0}^{n-1}{X}_{i}$
	Centroid	$\:{X}_{0}=\frac{\sum\:{X}_{oi}{A}_{i}}{{\sum\:A}_{i}}{Y}_{0}=\frac{\sum\:{Y}_{oi}{A}_{i}}{{\sum\:A}_{i}}$
	Irregularity index	$\:L=\:\frac{4\pi\:*A}{per}$
	Q_diameter_	$\:{Q}_{diameter}=\sqrt{\frac{4*A}{\pi\:}}$
	Contrast	$\:Contrast=\sum\:_{j}\sum\:_{i}{(j-i)}^{2}*P(j,\:i)$
	Homogeneity	$\:\text{H}\text{o}\text{m}\text{o}\text{g}\text{e}\text{n}\text{e}\text{i}\text{t}\text{y}=\sum\:_{j}\sum\:_{i}\frac{P(j,i)}{1+({j-i)}^{2}}$
	Dissimilarity	$\:Dissimilarity=\:\sum\:_{j,i=1}^{N}{P}_{j,i}|\:j-i\:|$
	Angular Second moment	$\:Angularsecondmoment=\:\sum\:_{j,i=1}^{N}{P}_{j,i}^{2}$
	Energy	$\:Energy=\:\sum\:_{j}\sum\:_{i}{P(j,i)}^{2}$
	Correlation	$\:Correlation=\:\frac{{\sum\:}_{j}{\sum\:}_{i}[ji*P\left(j,i\right)-{\mu\:}_{x}*{\mu\:}_{y}}{{\sigma\:}_{x}*{\sigma\:}_{y}}$
	Entropy	$\:Entropy=\:\sum\:_{j}\sum\:_{i}P\left(j,i\right)*\text{log}\left(P\left(j,i\right)\right)$

### Classification techniques

Because of their ability to automatically learn from picture data, CNNs have become the industry standard for categorization in image-based illness diagnosis. To further improve performance, hybrid models that integrate CNNs with machine learning classifiers, including SVMs, have also been investigated. CNNs can automatically learn spatial hierarchies in images, they are quite successful for image-based disease identification. When huge datasets are available, they perform better than conventional machine learning techniques like SVM and ANN^40^.

Research by^41^ demonstrated that by combining the advantages of both techniques, CNN, SVM models can perform better than standalone CNNs. SVMs, can improve classification when paired with CNNs because they can handle smaller datasets more accurately and with a reduced chance of overfitting.

ANNs have also been studied for crop disease classification, with substantial success, in addition to CNNs. However, because CNNs are better at capturing spatial hierarchies, they regularly perform better than ANNs in image-based tasks. If data augmentation is not applied properly, CNNs may readily overfitting to tiny or unbalanced datasets. Feature selection is increasingly using bio-inspired algorithms to better increase classification accuracy. To choose the most pertinent features for training classifiers, methods including Particle Swarm Optimization (PSO), Genetic Algorithms (GA), and WOA have been used^42,43–45^.

Recent research by^46,47^ integrated WOA with CNN for tomato leaf disease detection, showing the benefits of hybrid techniques, while showed the efficacy of PSO in optimizing feature selection for CNN based classification. These researches substantiate the possibility of enhancing classification accuracy and lowering computational cost by merging deep learning models with bio-inspired algorithms.

### Bio-inspired feature optimization

Bio-inspired optimization is an excellent exploration algorithm that can efficiently search huge feature spaces, while APSO enhances local exploitation and allows for faster convergence and more accurate solutions^48^.

### Research gap inference from the literature review

- Segmentation Limitations: Due of their high processing requirements, current segmentation approaches like U-Net and DeepLab struggle to be implemented in real-time, but they perform well in controlled situations. Furthermore, thresholding techniques are not very adaptable to changing environmental circumstances. This emphasizes the need for more effective segmentation techniques that can manage processing in real-time under a variety of environmental conditions.
- Issues with Feature Extraction: The intricate, multi-dimensional patterns found in diseased leaf photos are difficult for traditional texture-based approaches like GLCM to capture, especially in noisy or uneven illumination conditions. Though they have significant computational overhead and frequently need large datasets for efficient training, deep feature extraction models (such as VGG16 and ResNet) exhibit promise^49^. This suggests that a well-rounded strategy combining the strength of deep learning with lightweight feature extraction is required.
- Optimization Techniques: Although bio-inspired optimization algorithms like WOA and APSO have shown promise in enhancing feature selection, their hybrid applications frequently necessitate a great deal of parameter tweaking and might not translate well to other agricultural domains. More flexible optimization techniques that preserve good performance with minimal domain-specific tuning are needed.
- Classification Performance: CNNs are prone to overfitting on small datasets, notwithstanding their remarkable accuracy in detecting crop diseases. Although promising, hybrid approaches that combine CNN with SVM or ANN are still computationally costly and challenging to implement in real-time field applications^24,26^. A critical research gap is the creation of more effective classifiers or hybrid models that can function well with little datasets in real-time.

The above highlighted points are analysed and addressed by the proposed model, the following section gives the insights of the model with mathematical concept, algorithm and model diagram. For example by combining WOA with APSO, the improved APSO allows for faster convergence and better local exploitation by adjusting its search strategy in response to dynamic updates of particle positions and velocities. Nevertheless, there hasn’t been much research done on using hybrid bio-inspired algorithms with CNNs to automatically detect rice crop diseases. By enhancing feature selection, our suggested WOA_APSO algorithm seeks to close this gap and raise the CNN classifier’s overall performance.

## Proposed model

**Fig. 2:**
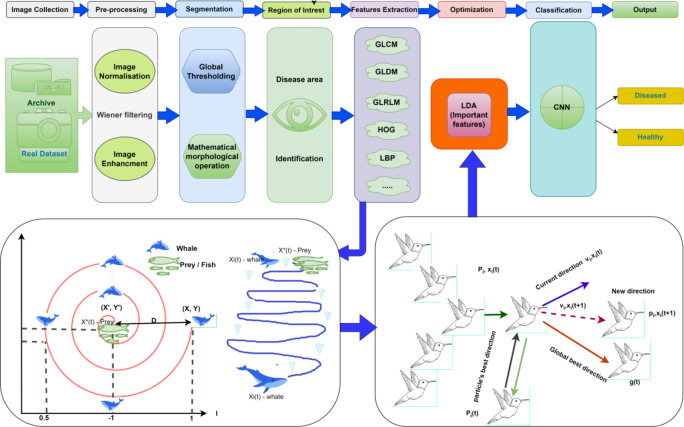
Architecture of proposed model.

The main goal of the work is to use an optimum feature selection-assisted classifier to classify the illnesses of rice leaves.The productions and quality of the crop will be adversely affected by any crop disease that affects the rice crop. With the goal to measures that can minimize rice yields loss improve rice quality and increase farmer income; it is important to accurately diagnose rice leaves disease. Currently most of the farmers must diagnosis and categorize disease by hand which takes lengthier. To get around this automated technique can be used to spot plant leaf disease. Data accessibility becomes a crucial component of CNN training since they are capable at classifying and identifying images. The detailed workflow of our proposed model is exhibited in Algorithm 1 and Fig. 2.

**Algorithm 1:**
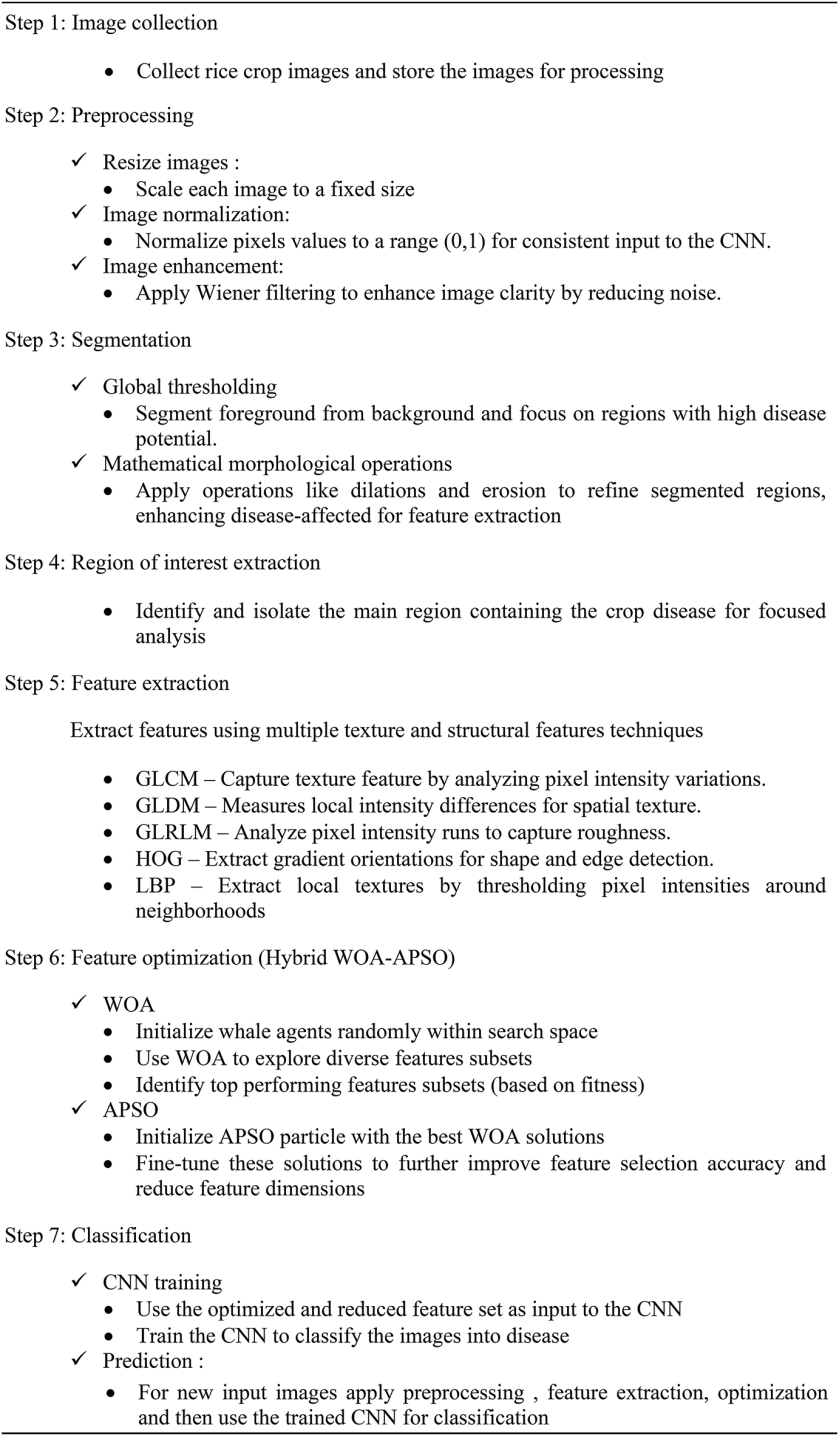
Hybrid WOA-APSO-based feature selection and CNN classification for rice crop disease detection.

The stages of the proposed computer-aided automatic diagnosis system methodology that are simulated and implemented are as follows the first step is images collected from Kaggle archive or real time dataset from filed. In the second step image preprocessing, it involves removing noise, enhancing image quality etc. The step 3 segment each input images followed by mathematical morphological operations step 5 and 6 is feature extraction and classification.

### Image collection

**Fig. 3:**
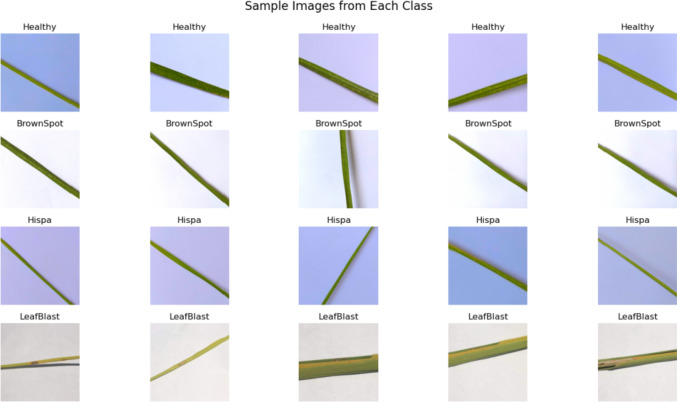
Each class sample images from archive.

The dataset contains 2096 rice crop images of four different classes which include healthy class. The dataset collected from kaggle archive for model training; the crop images stored in imaging archive undergo normalization. The Fig. 3 shows the sample images of our dataset. The images which were inputted mathematically expressed as in Eq. (1).

1 $$\:{R}_{ia}=\left\{{I}_{r1},\:{I}_{r2},{I}_{r3},\cdots\:\:{I}_{ri,}\right\}$$

where

R_i.a._ the input rice leaf archive.

I_r_ the leaf image present in the archive.

i total images in the archive.

### Preprocessing

**Fig. 4:**
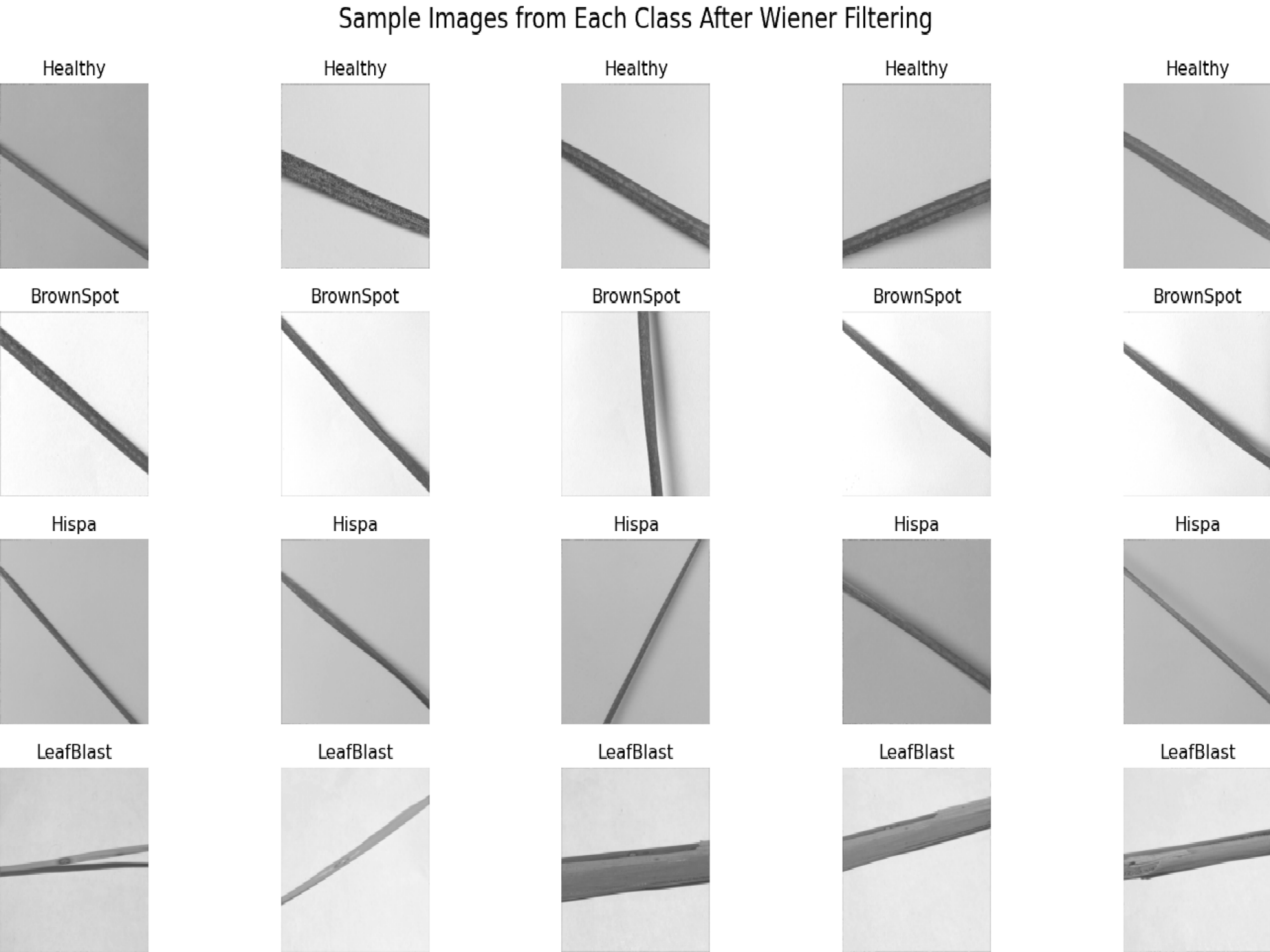
Sample images after applying wiener filter from each class.

Preprocessing, it is an optional process, it need when we acquire image from different environment, images is look upon as a crucial step in the process of identifying crop diseases and enhancing their quality. A wiener filter was applied to diagnosis the image with a minimum mean square error allows for image enhancement that is the statistical method for lessening the image’s blurring and smoothing effect. The Fig. 4 shows the result of after apply the wiener on the input image. The Wiener filter’s operation, as shown in mathematical model (2), is denoted by x[n].

2 $$\:x\left[n\right]=\:\sum\:_{i=0}^{n}{a}_{i}w[n-i]$$

where.

x[n] The main variable or signal of interest.

a_i_ Coefficients or weights associated with each term.

w[n − i] The shifted input or weighting function at step (n − i).

### Segmentation

The technique of dividing a picture into several sections, each with a distinct set of pixels, is known as image segmentation. The image is expected to be divided using the global thresholding technique based on the gray-level pixel intensity for threshold (T). Using (3), the segmented image obtained from global thresholding, can be expressed as s(x, y). In this case s(x, y) represents the image’s pixel value.

3 $$\:s\left(x,y\right)=\left\{\begin{array}{c}1\:if\:s\left(x,y\right)>threshold\left(T\right)\\\:0\:ifs\left(x,y\right)\le\:threshold\left(T\right)\end{array}\right.$$

where.

s(x, y) The value at coordinates(x, y)in the given space or matrix.

threshold(T ) The threshold value, represented by T, used for comparison.

1 Output when s(x, y) exceeds the threshold T.

0 Output when s(x, y) is less than or equal to the threshold T.

The segmented images is obtained by comparing the pixel intensity value with threshold value, the pixel intensity is greater than threshold values, the segmented image t(x, y) is obtained from the actual image, followed by segmentation mathematical morphological operations are estimated by applying a specific structuring element at every feasible location to smooth the region of interest,.

4 $$\:Erosion\::B\ominus\:S=\:\left\{A\:|\:{\left(S\right)}_{A}\:\subseteq\:B\right\}$$

5 $$\:Dilation\::B\:\oplus\:S=\:\left\{A\:|\:{\left(S\right)}_{A}\:\cap\:B\ne\:\:\varnothing\:\right\}$$

6 $$\:Opening:\:B\ominus\:S=\:B\ominus\:S\:\oplus\:BS$$

7 $$\:Closing\::B\:\ominus\:\:S=\:B\:\oplus\:S\:\oplus\:S$$

where.

B The input binary image or set being processed.

S The structuring element used for morphological operations.

⊖ The erosion operator, reduces the set B based on the structuring element S.

⊕ The dilation operator, which expands the set B using the structuring element S.

(S)_A_ The translated version of the structuring element S centered at A.

⊆ Denotes that (S)_A_ is a subset of B.

∩ Intersection operation.

∅ Represents the empty set.

**Fig. 5:**
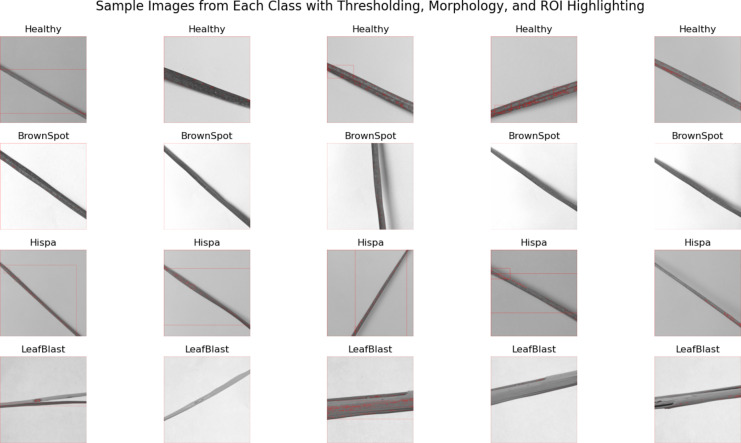
Sample images after applying thresholding, morphing and ROI.

This morphological operation mathematical expression is illustrated in (4), (5), (6), and (7), where B represents the binary image and S represents the structuring element.The erosion remove the unfinished part and prepare the image as thin one to accomplish the smoothening the image. The dilatation finishes the unfinished region of image boundaries and made thick to enhance the image. The Fig. 5 exhibits the sample images after applying thresholding, morphing and ROI. The ROI is highlighted with red box.

### Feature extraction

Feature extraction is a critical process for obtaining pattern information from segmented crop images. In the proposed approach, several techniques are employed to extract distinct geometrical, statistical, textural, and structural features from each segmented image. These include local binary pattern (LBP), histogram of oriented gradients (HOG), gray-level dependence matrix (GLDM), and gray-level co-occurrence matrix (GLCM). GLCM, a second-order statistical method, considers the spatial relationships between pairs of pixels. Higher-order statistical features, derived from clusters of continuous pixels with similar gray levels, are captured using the gray-level run length matrix (GLRLM). GLDM extracts features by calculating the absolute difference in gray levels between two pixels separated by a specified distance. HOG focuses on the structural aspects of the image by analyzing gradient orientations within localized regions using a feature descriptor. For rice crop images, LBP employs a shapebased operator to threshold neighbouring pixels based on the intensity of the central pixel. Table 5 outlines the features extracted from segmented images of damaged rice crops used for the analysis.

**Table 5:** Feature extracted by the feature extraction techniques.

Technique	Feature Count
GLCM	13
GLDM	6
GLRLM	5
HOG	1
LBP	10

### Feature optimization - Hybrid bio-inspired algorithm

Hybridisation of WOA with APSO may effectively enhance feature optimization by harnessing the advantages of two methods.

### Whale optimization algorithm

The working of WOA is social behaviour of humpback whales, specially the bubble-net feeding technique. The behaviors of WOA are encircling and bubble-net attacking, the feature subset selection is found by the WOA encircling and bubble-net attacking. It has couple of important phases that is exploitation and exploration. The WOA searching the prey at exploration, encircle the prey by using a bubble-net spiral method at exploitation phase. Spiral updating and prey encircling are updated by search agents randomly between spiral and prey encircling. The WOA has three important phases such as search for prey, encircling the prey and hunting^23^.

### Search for prey

**Fig. 6:**
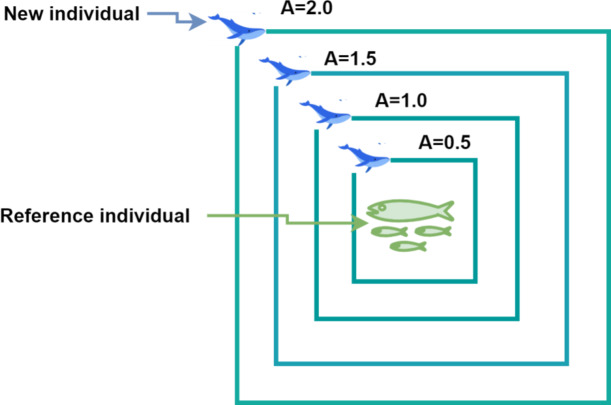
Search for the prey.

Whales search the prey in a random manner based on their relative position. The vector $\:\overrightarrow{A}$ plays a role in guiding this search process. The Fig. 6 illustrates the mathematical representation of how whales search for prey.

**Fig. 7:**
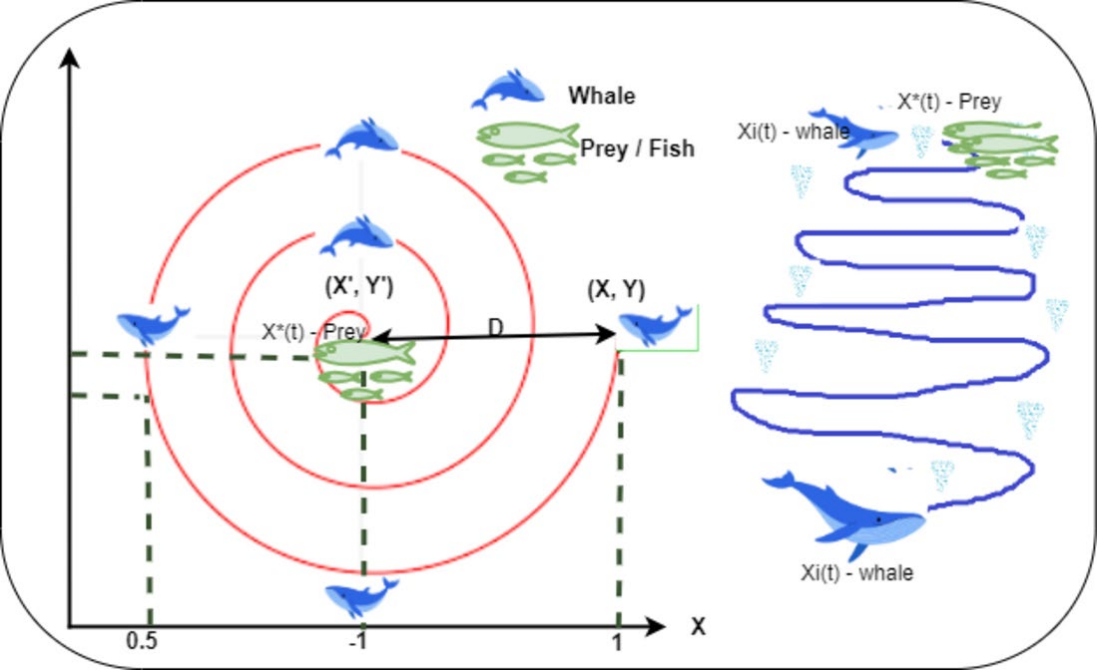
Whale optimization algorithm.

The $\:\overrightarrow{A}$ vector initialized by random values. If these values are greater than 1 or less than − 1, the search agent is directed to move farther away from a reference whale. During the exploration phase, unlike the exploitation phase, the position of the search agent is updated based on a randomly selected search agent rather than the best one identified so far. The algorithm $\:\left|\overrightarrow{A}\right|>1$, emphasize exploration and allow the WOA algorithm to perform a global search. The process continues to iterate $\:\overrightarrow{A}$ decreases linearly until $\:\left|\overrightarrow{A}\right|\le\:1$, the algorithm reach the stage of encircling the prey and hunting the fish. The Fig. 7 sows the whale optimization behavior.

$$\:\overrightarrow{\text{D}}=|\overrightarrow{\text{C}}.\overrightarrow{{\text{X}}_{\text{r}\text{a}\text{n}\text{d}}}-\overrightarrow{\text{X}}|$$

$$\:\overrightarrow{\text{X}}\left(\text{t}+1\right)=\overrightarrow{{\text{X}}_{\text{r}\text{a}\text{n}\text{d}}}-\overrightarrow{\text{A}}.\overrightarrow{\text{D}}$$

where

$\:\overrightarrow{{X}_{rand}}$ random position vector (a random whale) chosen from the current population.

$\:\overrightarrow{\text{D}}$ representing the difference between the current and random position.

$\:\overrightarrow{C}$ constant or coefficient vector that scales the difference between vectors.

$\:{\overrightarrow{X}}_{rand}$ A random position vector.

$\:\overrightarrow{X}$ The current position vector.

$\:\overrightarrow{A}$ A scaling factor or step size.

T The current time or iteration index.

The WOA algorithm starts with a set of random solutions, at each iteration search agents update their positions with respect to either a randomly chosen search agent or the best solution obtained so far. The parameter a is decreased from 2 to 0 in order to provide exploration and exploitation respectively. A random search agent is chosen when $\:\left|\overrightarrow{A}\right|>1$, while the best solution is selected when $\:\left|\overrightarrow{A}\right|<1$, for updating the position of the search agents. Depending on the value of probability, WOA is able to switch between either a spiral or circular movement. Finally the WOA algorithm is terminated by the satisfaction of a termination criterion.

The WOA can be considered a global optimizer because it includes exploration or exploitation ability. Furthermore the proposed hyper cube mechanism define a search space in neighborhood of the best solution and allows other search agents to exploit the current best record inside that domain adaptive variation of search vector $\:\overrightarrow{A}$ allows the WOA algorithm to smoothly transit between exploration and exploitation by decreasing $\:\overrightarrow{A}$ some iteration are devoted to exploration $\:\left|\overrightarrow{A}\right|<1$. Remarkably WOA includes only two main internal parameters to be adjusted (A and C). Although mutation and other evolutionary operations might have been included in the WOA formulation to fully reproduce the behavior of humpback whales we decided to minimize the amount of heuristic and the number of internal parameters thus implementing a very basic version of the WOA algorithm.

WOA begins with a set of randomly generated solutions. During each iteration search agents update their positions based either on a randomly chosen agent or the best solution identified so far. To facilitate both exploration and exploitation, the parameter a decreases linearly from 2 to 0. When $\:\left|\overrightarrow{A}\right|<1$, the algorithm opts for exploration by selecting a random search agent, whereas when $\:\left|\overrightarrow{A}\right|<1$, the best solution is used for position updates. Depending on the probability value, the WOA alternates between spiral and circular movements. The algorithm concludes once a predefined termination criterion is met.

WOA is considered a global optimizer due to its balanced exploration and exploitation capabilities. The proposed hypercube mechanism defines a search space around the best solution, enabling other agents to refine the search within this region. By adaptively varying the search vector A, WOA transitions smoothly between exploration and exploitation, with some iterations dedicated specifically to exploration $\:\left|\overrightarrow{A}\right|<1$. Notably, WOA relies on only two key internal parameters, A and C, simplifying its implementation. While additional mechanisms such as mutation could have been incorporated to better mimic the behavior of humpback whales, the algorithm was intentionally kept straight forward to minimize heuristics and internal parameters, resulting in a basic yet effective version of WOA.

### Encircling prey

Humpback whales exhibit a distinctive behavior known as bubble-net feeding, where they dive approximately 12 m below the water’s surface and create spiraling bubbles around their prey. They then swim upward toward the surface, herding the prey within the bubble net. This remarkable feeding strategy is unique to humpback whales. Each whale (agent) updates its position Xi (t) relative to the best-known position X*(t) (assumed as the prey). The distance between the whale and prey computed by (10) and (11).

10 $$\:\overrightarrow{D}=|\:\overrightarrow{C}.{\overrightarrow{X}}^{*}\left(t\right)-\overrightarrow{X}\left(t\right)|\:$$

11 $$\:\overrightarrow{{X}_{i}}\left(t+1\:\right)={\overrightarrow{X}}^{*}\left(t\right)-\overrightarrow{A}\:.\overrightarrow{D}$$

where

t the current iteration.

A, C are coefficient vector.

X* is the position vector of the best solution obtained so for.

X is the position vector.

$\:\overrightarrow{D}$ distance between whale i and best solution.

The vectors A and C are calculated as follows

12 $$\:\overrightarrow{C}=2.\overrightarrow{r}$$

13 $$\:\overrightarrow{A}=2.\overrightarrow{a}.\overrightarrow{r}-\overrightarrow{a}$$

$\:\overrightarrow{a}$ is the algorithm convergence factor linearly decreased from 2 to 0 over the course of iterations in both exploration and exploitation $\:a=2-\frac{2\:\times\:\:t}{{t}_{max}}$, $\:\overrightarrow{r}$ is a random vector in [0,1]

The position of a search agent is updated based on the location of the current best solution. By modifying the values of the $\:\overrightarrow{A}$ and $\:\overrightarrow{C}$ vectors, different positions around the best agent can be explored relative to the agent’s current location. The random vector r enables the search agent to reach and position itself within the search space between key points. Equation (10) allows any search agent to adjust its position within the vicinity of the best solution, effectively simulating the behavior of encircling prey. This concept can be extended to an n-dimensional search space, where agents navigate to positions around the best solution identified during the search process.

### Spiral updating – bubble-net attack

The bubble net attacking method has two sub tasks.

1. Shrinking encircling mechanism.

**Fig. 8:**
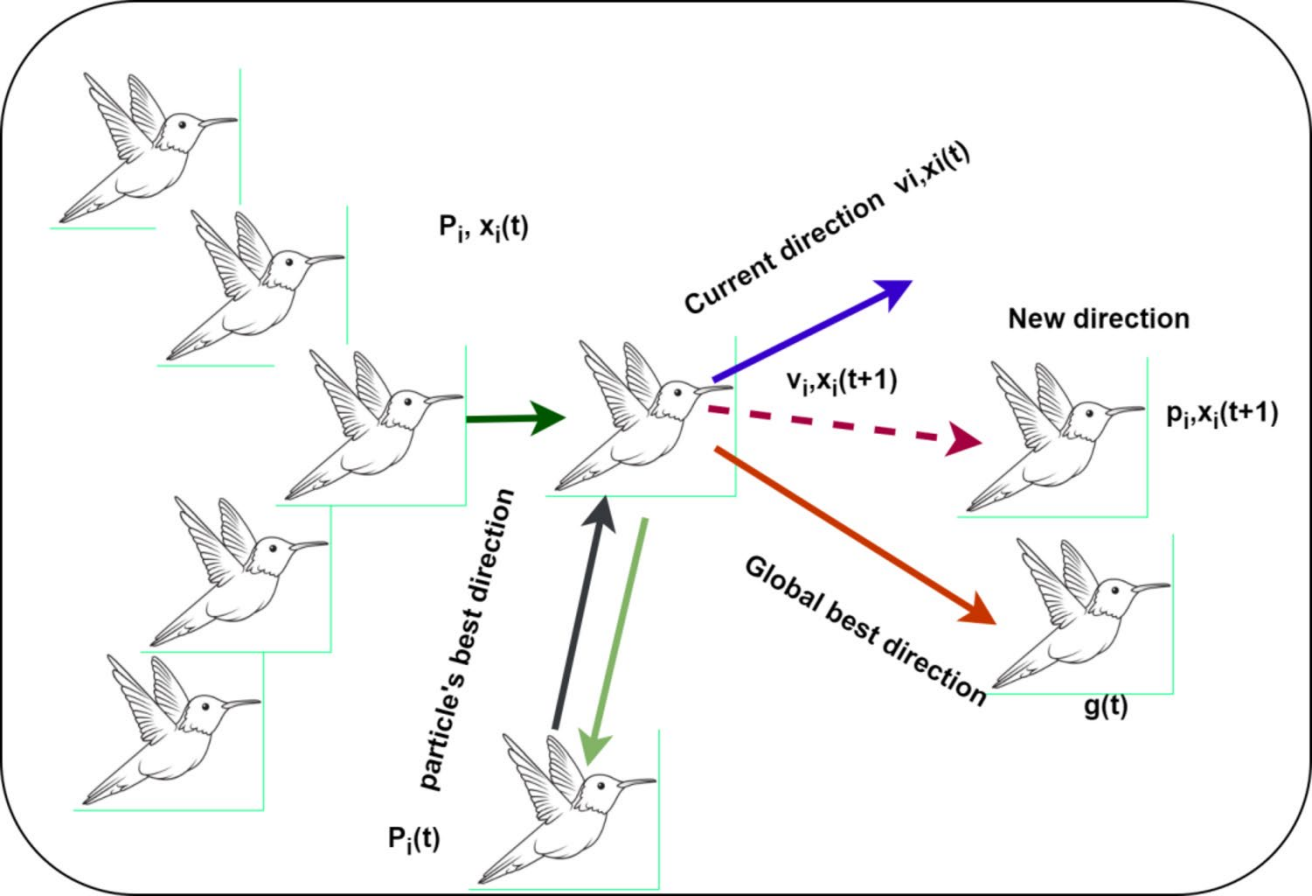
Adaptive particle swarm optimization algorithm.

The shrinking encircling mechanism is implemented by gradually reducing the value of $\:\overrightarrow{a}$ in the equation $\:\overrightarrow{A}=2.\overrightarrow{a}.\overrightarrow{r}-\overrightarrow{a}$. This results in $\:\overrightarrow{\text{A}}$ taking on random values within the range [− a, a], where a decreases linearly from 2 to 0 over the iterations. When $\:\overrightarrow{\text{A}}$ is assigned random values within the range [-1,1], the updated position of a search agent can lie anywhere between its original position and the position of the current best agent. The Fig. 8 illustrates the potential positions achievable along the path from x, y to X* when 0 < = A<=1.

2. Spiral updating position.

The spiral updating mechanism calculates the distance between the position of a whale at X and the prey at *X. To simulate the helical movement of humpback whales, a spiral equation is formulated between the positions of the whale and the prey. Whales adjust their positions along a spiral trajectory toward the optimal solution, as represented by Eq. (14).

14 $$\:{X}_{i}\left(t+1\right)=\overrightarrow{D}\:.{e}^{bl}.\text{cos}\left(2\pi\:l\right)+{X}^{*}\left(t\right)$$

D is $\:\overrightarrow{{D}^{{\prime\:}}}=|\overrightarrow{{X}^{*}}\left(t\right)-X\left(t\right)|$ and indicates the distance of the i^th^ whale of the prey (best solution obtained so far).

The parameter b is a constant that defines the shape of the logarithmic spiral, while l is a random value in the range [− 1, 1].

Humpback whales simultaneously move in a shrinking circular pattern and along a spiral path around the prey. To replicate this dual behavior, it is assumed that there is a 50% probability of selecting either the shrinking encircling mechanism or the spiral model for position updates during optimization. The corresponding mathematical representation captures this combined behavior.

Note that humpback whales swim around the prey within a shrinking circle and long a spiral shaped path simultaneously. To model this simultaneous behavior we assume that there is a probability of 50% to choose between both the shrinking encircling mechanism and the spiral model to update the positions of whales during optimization. The mathematical model is

15 $$\:\overrightarrow{X}\left(t+1\right)=\left\{\begin{array}{c}\overrightarrow{{X}^{*}}\left(t\right)-\overrightarrow{A}\:.\:\overrightarrow{D}\:\:\:\:\:\:\:\:\:\:\:\:\:\:\:\:\:\:\:\:\:\:\:if\:p<0.5\\\:\overrightarrow{{D}^{{\prime\:}}}.\:{e}^{bl}.\text{cos}\left(2\pi\:l\right)+\:\overrightarrow{{X}^{*}}\left(t\right)\:\:if\:p\ge\:0.5\end{array}\right.$$

where.

p is random number in [0,1].

### Switching mechanism

APSO imitates the social behavior of bird flocks. It enhances the standard Particle Swarm Optimization (PSO) by dynamically adjusting parameters, such as acceleration coefficients and inertia weight, based on the optimization state. Similar to birds adjusting their velocity and position relative to their current location, the optimal known position, and the flock’s best position, APSO particles adapt their movements accordingly. The particles in the swarm navigate the search space to identify the optimal solution. Each particle moves based on its own experience and the collective experience of the swarm. Every particle has three key attributes: position, velocity, and its previous best solution. The particle with the highest fitness value is referred to as the global best g_best_. The swarm consists of particles exploring and exchanging potential solutions, refining their search in pursuit of the global optimum. During the search, particles dynamically adjust their velocity based on their individual and collective flying experiences. Each particle maintains a record of its personal best solution (personal best- p_best_) and considers the global best solution (global best, g_best_) achieved by the swarm. The movement of a particle is influenced by its current position, velocity, and the distances to its (p_best_) and g_best_. Figure 8 illustrates the behavior of APSO along with its mathematical representation.

### APSO updates

Each particle adjusts its velocity and position in the search space is represented in (16)^50^ based on three main components such as inertia, cognitive and social components. The APSO adaption introduces a dynamic adjustment of these parameters based on current optimization states.

### Velocity update

16 $$\:{\text{v}}_{\text{i}}\left(\text{t+1}\right)\text{=w.}{\text{v}}_{\text{i}}\left(\text{t}\right)\text{+}{\text{c}}_{\text{1}}\text{.}{\text{r}}_{\text{1}}\text{.}\left({\text{p}}_{\text{i}}\left(\text{t}\right)\text{-}{\text{x}}_{\text{i}}\left(\text{t}\right)\right)\text{+}{\text{c}}_{\text{2}}\text{.}{\text{r}}_{\text{2}}\text{.}\left(\text{g}\left(\text{t}\right)\text{-}{\text{x}}_{\text{i}}\left(\text{t}\right)\right)$$

where.

vi(t + 1) Velocity of particle i at time t + 1.

W Inertia weight.

vi(t) Velocity of particle i at time t.

c1 and c2 Acceleration coefficients.

r1 and r2 Random numbers between 0 and 1.

pi(t) Personal best position of particle i at time t.

xi(t) Current position of particle i at time t.

g(t) Global best known position.

### Position updates

The position updates of particles are represented by (17).

17 $$\:{x}_{i}\left(t+1\right)=\:{x}_{i}\left(t\right)+{v}_{i}(t+1)$$

### Adaptive mechanisms and APSO process

APSO, w, c_1_, c_2_ adapt based on the swarm’s state increasing convergence speed and precision.

1. Get the input parameters like range min, max for each of the variables c1, c2, iteration counter = 0, V_max_, w_max,_ w_min_.
2. Initialize n number of population of particles of dimension d with random positions and velocities.
3. Increment iteration counter by one.
4. Evaluate the fitness function of all particles in the population, find particles best position, p_best_ of each particle and update its objective value. Similarly find the global best position g_best_ among all the particles and update its objective value.
5. If stopping criterion is met go to process 11. Otherwise continue.
6. Evaluate the inertia factor according to equation.

$$\:{\upomega\:}=\:{{\upomega\:}}_{\text{m}\text{a}\text{x}}-\left(\frac{\text{c}\text{u}\text{r}\text{r}\text{e}\text{n}\text{t}\_\text{i}\text{t}\text{e}\text{r}\text{a}\text{t}\text{i}\text{o}\text{n}}{\text{m}\text{a}\text{x}\_\text{i}\text{t}\text{e}\text{r}\text{a}\text{t}\text{i}\text{o}\text{n}}\right)({{\upomega\:}}_{\text{m}\text{a}\text{x}}-{{\upomega\:}}_{\text{m}\text{i}\text{n})}$$

so that each particles movement is directly controlled by its fitness value.

7. Update the velocity using the equation.

$\:{\text{v}}_{\text{i}}\left(\text{t+1}\right)\text{=w.}{\text{v}}_{\text{i}}\left(\text{t}\right)\text{+}{\text{c}}_{\text{1}}\text{.}{\text{r}}_{\text{1}}\text{.}\left({\text{p}}_{\text{i}}\left(\text{t}\right)\text{-}{\text{x}}_{\text{i}}\left(\text{t}\right)\right)\text{+}{\text{c}}_{\text{2}}\text{.}{\text{r}}_{\text{2}}\text{.}\left(\text{g}\left(\text{t}\right)\text{-}{\text{x}}_{\text{i}}\left(\text{t}\right)\right)$ and correct it using :

$$\:{v}_{ij}\left(t+1\right)=sign\left({v}_{ij}\left(t+1\right)\right)*\text{min}\left(\right|{v}_{ij}\left(t+1\right)\left)\right|,{V}_{jmax})$$

8. Update the position of each particle according to equation $\:{x}_{i}\left(t+1\right)=\:{x}_{i}\left(t\right)+{v}_{i}(t+1)$ if the new position goes out of range sit it to the boundary value using equation :

$$\:{x}_{ij}\left(t+1\right)=\text{min}\left({x}_{ij}\left(t+1\right)\right),{range}_{jmax,}$$

$$\:{x}_{ij}\left(t+1\right)=\text{max}\left({x}_{ij}\left(t+1\right)\right),{range}_{jmin}$$

9. The elites are inserted in the first position of the new population in order to maintain the best particle found so far.
10. For every 5 generations this F_best_, new value is compared with the F_best_, old, if there is no noticeable change then re-initialize K % of the population. Go to process 3.
11. Output the g_best_ particle and its objective value.

### WOA-APSO

The hybrid WOA_APSO has two primary components. WOA is exploration and APSO exploitation, the mathematical for each component is brief explained in the above paragraph. The precision optimal and broader solution is covered by the WOA for exploration and APSO for fine tuning hybrid approach. After WOA converges on promising regions APSO is used to refine the feature subset selection within these regions. The hybrid WOA_APSO algorithm begins with a random solution. The processes of hybrid optimization are brief as follows.

Step 1: Define initial population of features including the positions of both APSO (birds/particles) and WOA (search agents) randomly.

Step 2: Exploration phase here to focus on different regions of the search space by WOA encircling behavior, to optimize the feature set we calculate fitness of each whales and update the whale positions according to the best solutions found, leading them closer to an optimal feature subset.

Step 3: Exploitation phase here fins tuning by APSO, The APSO get current best solution from the WOA as the beginning positions for APSO particles, then update the particle velocities and positions by APSO adaptive rules to further refine the feature subset, ensuring convergence to a more optimal solution.

Step 4: Convergence check, set the convergence constraint or condition. It the convergence is reached stop the process else the alternate between WOA and APSO till convergence reached.

### Pseudo code - WOA_APSO

1. Initialize the population for a search agents randomly.

Iteration initial value t=0, Maxiteration=100.

Coefficient a=2-t*((2) /Maxiteration.

Coefficient A=2*a*r1-a.

Coefficient C=2*r2.

Random value =r.

No of search agents = 5 or initialize population size *n*= 5.

2. Calculate the fitness value for each search agent.

3. Choose best search agent.

4. While(t< MaxT).

5. Update w, a,A, C,l and p for each search agent.

6. For each search agent.

7. if1(*p*<0.5).

8. if2(|A|>1) select random agent and update position

$$\:X\left(t+1\right)={X}_{random}\left(t\right)-A*D$$

9. elseif2(|A|<1) update position of agent.

10. elseif2 $$\:X\left(t+1\right)=\left\{\begin{array}{c}w.{X}^{*}\left(t\right)=A.D\:,\:p<0.5\\\:{D}^{{\prime\:}}.{e}^{bl}.\text{cos}\left(2\pi\:l\right)+w.{X}^{*}\left(t\right),\:p>0.5\end{array}\right.$$

11. elseif1(p>=0.5) update.

12. calculate fitness for each agent.

13. update optimal solution.

14. increment counter t=t+1.

15. returned best search and its fitness value.

**Table 6:** Hybrid WOA-APSO parameters and values.

Parameter	Values
Lower bound (Lb)	1
Upper bound (Ub)	160
Dimension	20
Maximum Iteration	35
Gamma	0.5
Search agents no	25

In the proposed work we apply this concept to search for optimal features from the extracted features. The objective of the hybrid WOA_APSO is to select the features optimally, which improves the system’s overall classification efficiency. The WOA_APSO accepts the extracted feature vector containing attributes like edges, texture patterns, contrast, spatial attributes, etc., as input. The WOA_APSO algorithm starts with initializing a maximum number of iteration and population sizes that is number of search agents in which each swarm / whale indicates the potential image feature subset. After initialization the fitness value of each feature subset was determined based on the defined objective function that maximizes classification performance by reducing data dimension, training time and over fitting issues, then the fitness value of the individual feature subset swarm /whale is estimated based on its effectiveness on classification performance (Table 6).

### Classification

A deep learning classification method for training and testing the learning network is the convolutional neural network. The neural network is composed of three densely connected layers with activation functions that link one neuron to another. The weights and deltas are updated using the backpropagation technique, which has a 0.001 learning rate. To find the ideal combination for figuring out how resilient the experiment is, testing is done on a variety of criteria. The following are the different parameters:^5,10,15,20^ are the layer neurons, relu, softmax are the activation functions; batch size is 1,2,3; validation split is 0.1, 0.2,0.3; learning rate is 0.1, 0.01,0.001; and epochs are 10, 20, 40, 60, 80, 100, and 200. The CNN’s parameters and values are shown in Table 7.

**Table 7:** CNN parameters and values.

CNN model Parameters	Values
Layer	5
Activation function	Relu, Softmax
Optimizer	Adam
Loss	Categorical_crossentropy
Epochs	10
Validation split	0.2

### CNN structure

The CNN will be made up of several layers that are intended to learn from the features that are extracted:

The CNN input layer accepts the 224 × 224 pixel scaled photos and Convolutional Layers to extract hierarchical characteristics, apply several filters. For example, whereas deeper layers learn complex structures, the initial layer may only learn edges. Activation function to introduce non-linearity, use the rectified linear unit (ReLU). Pooling layers to minimize dimensionality while preserving significant characteristics, max pooling will be utilized. Fully connected layer provides the final categorization into groups like Leaf Blast, Brown Spot, Hispa, and Healthy by combining features. Assessment of performance to guarantee robustness, the model will be assessed using a range of performance metrics: The proportion of accurately anticipated cases to all instances is known as accuracy. Sensitivity (Recall): Evaluates how well the model detects affirmative cases. Specificity: Indicates how well the model can detect negative cases. Computational cost: Examine how long and what resources the model required to train and test.

### Results and discussion

To assess the effectiveness of the experiments, we use 2096 rice crop image kaggele dataset which has four classes. The WOA and APSO are contrasted with the optimal threshold value obtained from the WOA_APSO algorithm. WOA_APSO, WOA, and APSO have reached their respective threshold values of 1.16, 2.09, and 1.9. Thus, the suggested hybrid WOA_APSO algorithm with a bio-inspired design provides accurate information-optimized feature subsets. Table 8 displays the comparative performance analysis of several classification techniques.

**Table 8:** Performance comparison of classification technique.

Model	Accuracy in %	Sensitivity	F1 Score in %
Proposed Hybrid	97.5	95	96
ANN	88	85	89
CNN	92	90	94
SVM	85	82	87

**Fig. 9:**
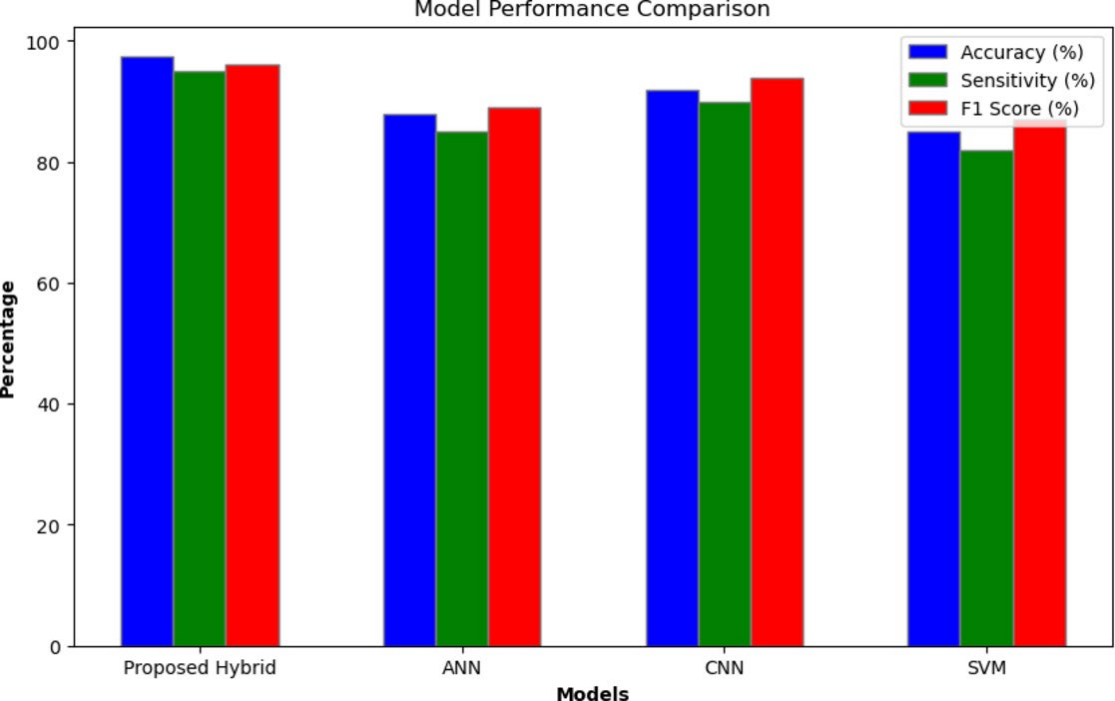
Performance analysis.

The Fig. 9 shows the performance measures of rice crop diseases are compared with the different classification algorithms.

The evaluation analysis parameters used for determining the effectiveness of the model are accuracy, sensitivity and specificity shown in (18), (19) and (20) respectively.

18 $$\:Accuracy=\:\left[\frac{TP+TN}{TOTAL}\right]\:X\:100$$

19 $$\:Sensitivity=\:\left[\frac{TP}{TP+FN}\right]\:X\:100$$

20 $$\:Specificity=\:\left[1-FPR\right]\:X\:100$$

where

TP shows that number of images correctly classified.

FN shows that the number of images incorrectly classified.

FPR the incorrect images classified correctly.

**Fig. 10:**
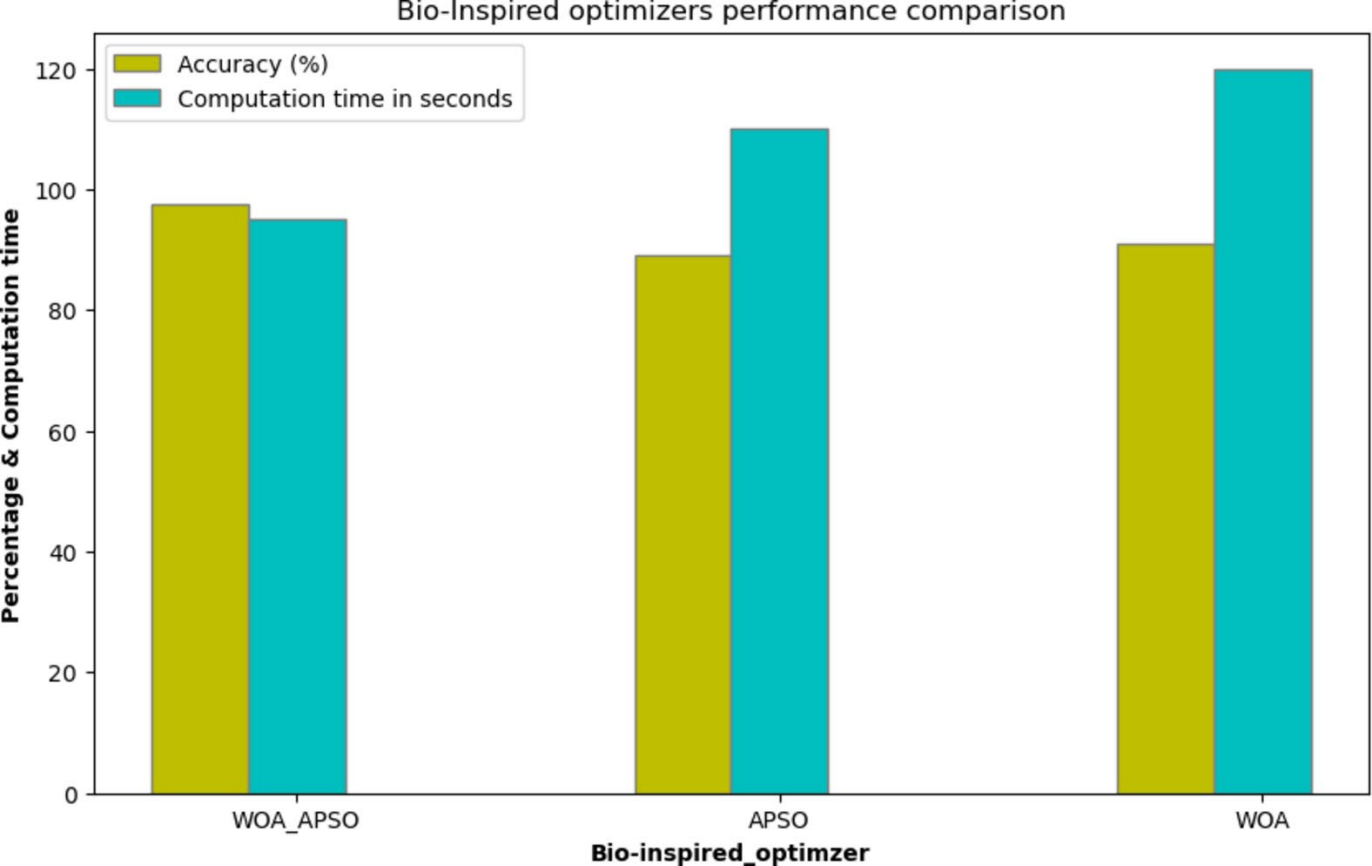
Impact of feature optimization.

**Fig. 11:**
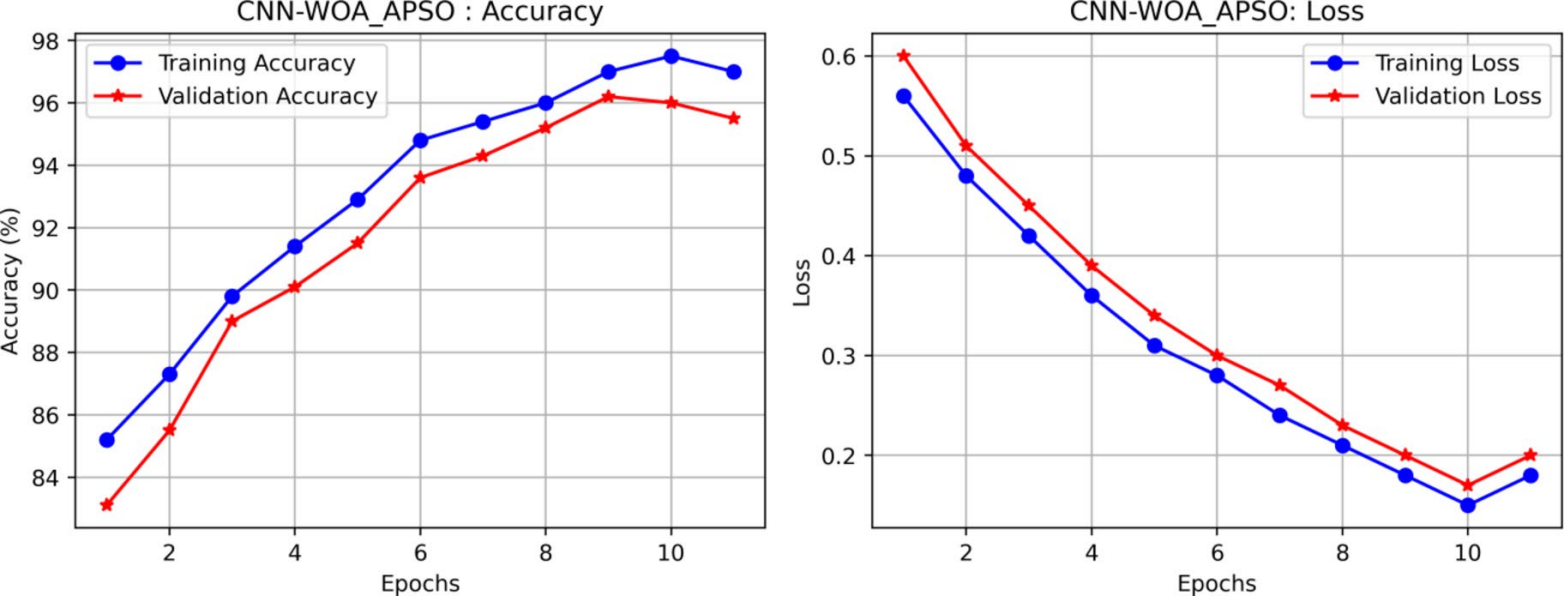
Model accuracy and loss C curve.

Table 9 shows the APSO, WOA and WOA_APSO the computational time. The APSO and WOA take more time when compare to WOA_APSO. By contrasting the various classification methods, hybrid WOA_APSO with CNN model performance metrics are determined. The model’s efficacy was assessed using the evaluation analysis parameters of accuracy, sensitivity, and specificity, as presented in (18), (19) (20) in that order. Figure 10 shows the APSO, WOA and WOA_APSO the computational time and accuracy. The APSO and WOA take more time when compare to WOA_APSO. Figure 11 represents the model accuracy and loss function.

**Table 9:** Comparison of computation time and accuracy of feature optimizers.

Model	Computation Time (s)	Accuracy
APSO	110	0.89
WOA	120	0.91
WOA_APSO	95	0.975

## Conclusion

In order to increase the accuracy of rice crop disease detection and take preventative action against rice crop yield loss, also tries to overcome current issues in the field, such as computational efficiency, and generalization, by concentrating on robust feature extraction and selection. By providing rice farmers with insights into automated disease monitoring and intervention options, this research advances precision agriculture. We have proposed a unique method for early detection, diagnosis, and prediction in this study. We used a hybrid WOA_APSO algorithm in this instance. The diseased region of crop image is partitioned and segmented using an image preprocessing and segmentation approach. Our goal is to address the computational and generalization issues raised by earlier research by combining CNN with a hybrid bio-inspired WOA_APSO method. Even with lesser datasets, our method will improve feature selection and classification performance while guaranteeing the system’s scalability and adaptability for real-time field applications. Furthermore, we anticipate greatly enhancing the resilience and effectiveness of rice crop disease detection by combining cutting-edge image processing techniques and striking a balance between exploration and exploitation in optimization. The many elements are taken out in order to compile statistical data and analyze it, which helps in decision-making. By utilizing a hybrid WOA_APSO algorithm, the suggested state-of-the-art method offers a superior consolidated optimal dimension of features selection grouping approach. When compared to artificial neural networks and support vector machines, the convolutional neural network classification method performs better, offering an accuracy of 97.5%. Our suggested hybrid WOA_APSO algorithm, which is integrated with CNN, seeks to overcome these difficulties by increasing the effectiveness of feature selection and improving classification accuracy, even on small, unbalanced datasets. It also makes sure that the system is computationally feasible for real-time use in a variety of field conditions. Future studies could look into real time implementation, linking the model to internet of things gadgets to enable on going observation, Transfer learning applying transfer learning strategies to modify the model for different crops illnesses and multimodal data integration, increasing prediction accuracy by utilizing other data sources, like weather patterns and soil health.

## Data Availability

The datasets generated and/or analysed during the current study are available in the Kaggle repository, https://www.kaggle.com/datasets/minhhuy2810/rice-diseases-image-dataset.

## References

[1] Asma, J., Subrahmanyam, D. & Krishnaveni, D. The global lifeline: A staple crop sustaining two thirds of the world’s population. *Agric. Archives* **2**(3), (2023).

[2] Garcia Amaro, E. et al. Use of computer vision techniques for recognition of diseases and pests in tomato plants. *Computacion Y Sistemas* **28**(2), 709–723 (2024).

[3] Tyagi, S., Reddy, S., Anand, R. & Sabharwal, A. Enhancing rice crop health: a light weighted CNN-based disease detection system with mobile application integration. *Multimedia Tools Appl.* **83**, 48799–48829 (2024).

[4] Shrotriya, A., Sharma, A., Prabhu, S. & Bairwa, A. An approach towards classifying Plant-Leaf diseases and comparisons with the conventional classification. *IEEE Access.* **12**, 117379–117398 (2024).

[5] Javaid, M., Haleem, A., Khan, I. & Suman, R. Understanding the potential applications of artificial intelligence in agriculture sector. *Adv. Agrochem*. **2**, 15–30 (2023).

[6] Shafi, U. et al. Wheat yellow rust disease infection type classification using texture features. *Sensors* **22**, 146 (2021). 35009689 10.3390/s22010146 PMC8747460

[7] Rahimi-Ajdadi, F. & Mollazade, K. Image deblurring to improve the grain monitoring in a rice combine harvester. *Smart Agricultural Technol.* **4**, 100219 (2023).

[8] Haridasan, A., Thomas, J. & Raj, E. Deep learning system for paddy plant disease detection and classification. *Environ. Monit. Assess.* **195**, 120 (2023). 10.1007/s10661-022-10656-x 36399232

[9] Suseno, J., Azhar, Y. & Minarno, A. The Implementation of Pretrained VGG16 Model for Rice Leaf Disease Classification using Image Segmentation. Kinetik: Game Technology, Information System, Computer Network, Computing, Electronics, And Control. pp. 499–506 (2023).

[10] Odukoya, O., Aina, S. & Dégbéssé, F. A. Model for detecting fungal diseases in cotton cultivation using segmentation and machine learning approaches. *Int. J. Adv. Comput. Sci. Appl.* **14**(1), 628–636 (2023).

[11] Xie, X. et al. A novel feature selection strategy based on salp swarm algorithm for plant disease detection. *Plant. Phenomics*. **5**, 0039 (2023). 37228513 10.34133/plantphenomics.0039 PMC10204742

[12] Shafik, W., Tufail, A., Namoun, A., De Silva, L. & Apong, R. A systematic literature review on plant disease detection: motivations, classification techniques, datasets, challenges, and future trends. *Ieee Access.* **11**, 59174–59203 (2023).

[13] Erdem, F. & Bayrak, O. Evaluating the effects of texture features on Pinus sylvestris classification using high-resolution aerial imagery. *Ecol. Inf.* **78**, 102389 (2023).

[14] Khan, B. et al. Bayesian optimized multimodal deep hybrid learning approach for tomato leaf disease classification. *Sci. Rep.* **14**, 21525 (2024). 39277634 10.1038/s41598-024-72237-x PMC11401875

[15] Jasrotia, S., Yadav, J., Rajpal, N., Arora, M. & Chaudhary, J. Convolutional neural network based maize plant disease identification. *Procedia Comput. Sci.* **218**, 1712–1721 (2023).

[16] Attri, I., Awasthi, L., Sharma, T. & Rathee, P. A review of deep learning techniques used in agriculture. Ecological Informatics. p. 102217 (2023).

[17] Din, N. et al. RiceNet: A deep convolutional neural network approach for classification of rice varieties. *Expert Syst. Appl.* **235**, 121214 (2024).

[18] Muthukumaran, S., Geetha, P. & Ramaraj, E. Multi-Objective optimization with artificial neural network based robust paddy yield prediction model. *Intell. Autom. Soft Comput.* **35**(1), 215–230 (2023).

[19] Dogan, M. & Ozkan, I. Determination of wheat types using optimized extreme learning machine with metaheuristic algorithms. *Neural Comput. Appl.* **35**, 12565–12581 (2023).

[20] Kumar, A., Ahmad, F. & Alam, B. Hybrid bio-inspired computing in medical image data analysis: A review. *Intell. Decis. Technol.*, 1–18 (2024).

[21] Mohanty, S., Hughes, D. & Salathé, M. Inference of plant diseases from leaf images through deep learning. *Front. Plant. Sci.* **7**, 1419 (2016). 27713752 10.3389/fpls.2016.01419 PMC5032846

[22] Ramcharan, A. et al. Deep learning for image-based cassava disease detection. *Front. Plant Sci.* **8**, 1852 (2017). 29163582 10.3389/fpls.2017.01852 PMC5663696

[23] Mirjalili, S. & Lewis, A. The Whale optimization algorithm. *Adv. Eng. Softw.* **95**, 51–67 (2016).

[24] Zhang, Q., Liu, Y., Gong, C., Chen, Y. & Yu, H. Applications of deep learning for dense scenes analysis in agriculture: A review. *Sensors* **20**, 1520 (2020). 32164200 10.3390/s20051520 PMC7085505

[25] Chen, S. et al. An approach for rice bacterial leaf streak disease segmentation and disease severity Estimation. *Agriculture* **11**, 420 (2021).

[26] Sun, C., Zhou, X., Zhang, M. & Qin, A. SE-Vision transformer: hybrid network for diagnosing sugarcane leaf diseases based on attention mechanism. *Sensors* **23**, 8529 (2023). 37896622 10.3390/s23208529 PMC10611343

[27] Zhang, Y., Wang, J., Li, X., Huang, S. & Wang, X. Feature selection for High-Dimensional datasets through a novel artificial bee colony framework. *Algorithms* **14**, 324 (2021).

[28] Prabukumar, M., Agilandeeswari, L. & Ganesan, K. An intelligent lung cancer diagnosis system using cuckoo search optimization and support vector machine classifier. *J. Ambient Intell. Humaniz. Comput.* **10**, 267–293 (2019).

[29] Mittal, H. & Saraswat, M. An automatic nuclei segmentation method using intelligent gravitational search algorithm based superpixel clustering. *Swarm Evol. Comput.* **45**, 15–32 (2019).

[30] Zou, K., Chen, X., Wang, Y., Zhang, C. & Zhang, F. A modified U-Net with a specific data argumentation method for semantic segmentation of weed images in the field. *Comput. Electron. Agric.* **187**, 106242 (2021).

[31] Ahmad, N. et al. Leaf image-based plant disease identification using color and texture features. *Wireless Pers. Commun.* **121**, 1139–1168 (2021).

[32] Shaukat, A. et al. Textural and geometrical features based approach for identification of individuals using palmprint and hand shape images from multiple multimodal datasets. *J. Test. Eval.* **46**, 2281–2298 (2018).

[33] Sachar, S. & Kumar, A. Automatic plant identification using transfer learning. *IOP Conf. Series: Mater. Sci. Eng.* **1022**, 012086 (2021).

[34] Tholkapiyan, M. et al. Performance analysis of rice plant diseases identification and classification methodology. *Wireless Pers. Commun.* **130**, 1317–1341 (2023).

[35] Kurniati, F. T., Sembiring, I., Setiawan, A., Setyawan, I. & Huizen, R. R. GLCM-Based Feature Combination for Extraction Model Optimization in Object Detection Using Machine Learning. *J. Ilm. Tek. Elektro Komput. Dan Inform.* **9**(4), 1196–1205 (2024).

[36] Sarode, K., Savdekar, R. & Chaudhari, T. Texture feature analysis of an image using Gray level Co-Occurrence matrix. *Int. J. Novel Res. Dev.* **7**, 139–143 (2022).

[37] Kabir, M., Unal, F., Akinci, T., Martinez-Morales, A. & Ekici, S. Revealing GLCM metric variations across a plant disease dataset: A comprehensive examination and future prospects for enhanced deep learning applications. *Electronics* **13**, 2299 (2024).

[38] Mutlag, W., Ali, S., Aydam, Z. & Taher, B. Feature extraction methods: a review. Journal of Physics: Conference Series. 1591, 012028 (2020).

[39] Archana, K. et al. A novel method to improve computational and classification performance of rice plant disease identification. *J. Supercomputing* **78**, 8925–8945 (2022).

[40] Jiang, F., Lu, Y., Chen, Y., Cai, D. & Li, G. Image recognition of four rice leaf diseases based on deep learning and support vector machine. Computers and Electronics in Agriculture. 179 p. 105824 VOLUME 11, 2023 15 Author et al.: Preparation of Papers for IEEE TRANSACTIONS and JOURNALS (2020).

[41] Ali, A. & Mallaiah, S. Intelligent handwritten recognition using hybrid CNN architectures based-SVM classifier with dropout. *J. King Saud University-Computer Inform. Sci.* **34**, 3294–3300 (2022).

[42] Moreno-Revelo, M., Guachi-Guachi, L., Gómez-Mendoza, J. & Revelo-Fuelagán, J. Peluffo-Ordóñez, D. Enhanced convolutional-neural-network architecture for crop classification. *Appl. Sci.* **11**, 4292 (2021).

[43] Sankareshwaran, S. P. et al. Optimizing rice plant disease detection with crossover boosted artificial hummingbird algorithm based AX-RetinaNet. *Environ. Monit. Assess.* **195**, 1070. 10.1007/s10661-023-11612-z (2023). 37610473 10.1007/s10661-023-11612-z

[44] Pandi, S. S. et al. Rice plant disease classification using dilated convolutional neural network with global average pooling. *Ecol. Model.* **474**, 110166 (2022).

[45] Padhi, J. et al. *Enhancing Paddy Leaf Disease Diagnosis-a Hybrid CNN Model Using Simulated Thermal Imaging*100814 (Smart Agricultural Technology, 2025).

[46] Aguerchi, K., Jabrane, Y., Habba, M. & El Hassani, A. A CNN hyperparameters optimization based on particle swarm optimization for mammography breast Cancer classification. *J. Imaging*. **10**, 30 (2024). 38392079 10.3390/jimaging10020030 PMC10889268

[47] Thanammal Indu, V. & Suja Priyadharsini, S. Crossover-based wind-driven optimized convolutional neural network model for tomato leaf disease classification. *J. Plant Dis. Prot.* **129**, 559–578 (2022).

[48] Li, Z., Tam, V. & Yeung, L. An adaptive multi-population optimization algorithm for global continuous optimization. *IEEE Access.* **9**, 19960–19989 (2021).

[49] Singh, P., Chaudhury, S., Panigrahi, B. & Hybrid, M. P. S. O. C. N. N. Multi-level particle swarm optimized hyperparameters of convolutional neural network. *Swarm Evol. Comput.* **63**, 100863 (2021).

[50] Oubelaid, A. et al. Intelligent torque allocation based coordinated switching strategy for comfort enhancement of hybrid electric vehicles. *IEEE Access.* **10**, 58097–58115 (2022).

